# High populations near fossil fuel energy infrastructure across the supply chain and implications for an equitable energy transition

**DOI:** 10.1088/1748-9326/ae0da6

**Published:** 2025-11-17

**Authors:** Jonathan J Buonocore, Fintan A Mooney, Erin J Campbell, Brian Sousa, Breanna van Loenen, M Patricia Fabian, Amruta Nori-Sarma, Mary D Willis

**Affiliations:** 1 Department of Environmental Health, School of Public Health, Boston University, Boston, MA, United States of America; 2 Institute for Global Sustainability, Boston University, Boston, MA, United States of America; 3 Department of Epidemiology, School of Public Health, Boston University, Boston, MA, United States of America; 4 Department of Environmental Health Sciences, Columbia Mailman School of Public Health, New York, NY, United States of America

**Keywords:** energy infrastructure, population exposure, fossil fuels, exposure assessment, supply chains, oil and gas

## Abstract

Fossil fuel energy infrastructure poses health risks for local communities, primarily due to the presence of air pollution emissions and other hazards. There is also evidence of racial/ethnic disparities in the siting of this infrastructure for select components. However, population counts and demographic composition near fossil fuel energy infrastructure have not been systematically characterized across all types, supply chain stages, and predominant fuel types. Here, we construct a dataset of 25 elements of fossil fuel energy infrastructure and characterize the populations living near this infrastructure (defined as within 800 m [∼0.5 mile] or 1.6 km [∼1 mile]). We estimated that 46.6 million people in the contiguous U.S., representing 14.1% of the population, live within 1.6 km of at least one piece of energy infrastructure, with racial/ethnic disparities observed across nearly all stages of the supply chain. End use infrastructure has the most people residing within 1.6 km, with 20.9 million people, followed by extraction (20.3 million), and storage (6.16 million). Storage infrastructure has an average of ∼2,900 people living within 1.6 km of each element; end use infrastructure has an average of 1,900 people residing within 1.6 km of each element; extraction infrastructure has an average of 17 people residing within 1.6 km of each element. Almost 90% of the population near end use, transportation, refining, and storage infrastructure are in urban areas. Our results represent a substantial population in the U.S. that is potentially exposed to hazards that are not well-characterized, with unknown cumulative impacts, and which constitute a major environmental justice issue.

## 1. Introduction

Fossil fuel energy infrastructure emits pollutants with well-documented impacts on public health and environmental justice [1, 2]. Many elements of fossil fuel energy infrastructure substantially contribute to regional air pollution (e.g. PM_2.5_, ozone) and the consequent public health impacts [3–6]. This industry also produces local-scale environmental hazards that are not well-characterized for the communities who host this infrastructure, such as air toxics [7–10], water contamination [11–15], induced vehicular traffic [16], noise [17], and social change [15, 18]. Understanding the cumulative impacts on public health for communities exposed to several types of fossil fuel infrastructure may be exceptionally challenging, as the range of exposure pathways and associated stressors may interact in non-linear ways [19]. For instance, epidemiological studies have found associations between residential proximity to fossil fuel extraction (e.g. coal, oil, gas) and a wide array of health outcomes [20, 21], including reduced birth weight [22–24], pediatric asthma [25–27], childhood leukemia [28], and depression or anxiety symptoms [29–31]. Furthermore, an emerging body of literature shows that the communities who host this infrastructure are often racially marginalized, lower-income, and/or environmentally burdened [2, 11, 18, 32, 33]. However, the co-occurrence of exposure to fossil fuel infrastructure remains largely unquantified to date.

There is evidence of public health hazards across the entire supply chain of fossil fuels, including processes in the ‘upstream’ (e.g. oil and gas extraction, coal mining), ‘midstream’ (e.g. physical transportation through pipelines, refining or processing, and storage), and ‘downstream’ (e.g. combustion of fuels to generate electricity, and distribution of gas to local consumers) [2, 34–36]. For example, power plants produce environmental stressors including precursors to major regional air pollutants [35, 37], pattern with adverse health outcomes in nearby communities [27, 38–41], and present environmental justice issues in siting [42]. Gas pipeline compressor stations emit hazardous air pollutants [43–46], and are often sited near marginalized communities [46]; their emissions have been associated with increased mortality rates [45]. Underground gas storage facilities have at least 50 000 people living within 200 m, potentially creating acute safety hazards from leaks and other events [47, 48]. Proximity to refining has also been associated with risk of stroke [49] and cancer [50]. Opportunity for exposure to hazardous air pollutants persist throughout all phases of the production life cycle [49, 51–57].

Despite evidence of health impacts and presence of hazards across the full supply chain of fossil fuels, populations living near all elements of fossil fuel supply chains in the U.S. have not yet been systematically characterized. Here, we quantify and characterize populations residing near fossil fuel energy infrastructure in the U.S. at two buffer distances—800 m and 1.6 km (approximately 0.5 and 1 mile; selected to align with previous literature [58]) using a novel integrate dataset of 25 types of fossil fuel infrastructure (>1.2 million elements) (tables 1 and S1). We estimate and compare populations living near various fossil fuel infrastructure types within these two buffer distances, including co-occurring population exposures and potential demographic inequalities.

**Table 1.** Types of fossil fuel energy infrastructure within the [EI]^3^ database.

Fossil fuel energy infrastructure (*N* = *25*)	Data source	Phase of supply chain	Description	Count
Coal & Biomass power plants	HFILD	Downstream	Power plants that burn a mix of coal and biomass	13
Coal power plants	HIFLD	Downstream	Power plants that burn coal	529
Coal surface mines	U.S. EIA	Upstream	Surface coal mines in the U.S.	337
Coal underground mines	U.S. EIA	Upstream	Underground coal mines in the U.S.	174
Crude oil railroad terminals	U.S. EIA	Transportation	Rail terminals that handle the loading and unloading of crude oil	94
Gas & Biomass power plants	HIFLD	Downstream	Power plants that burn a mix of gas and biomass	20
Gas & Municipal solid Waste power plants	HIFLD	Downstream	Power plants that burn a mix of gas and municipal solid waste	1
Gas compressor stations	U.S. Energy Information Administration (EIA)	Transportation	Compressor stations that pressurize gas along pipelines	1,866
Gas import/export terminals	HIFLD	Transportation	Gas import/export facilities along the borders between the Continental United States, Canada, and Mexico	31
Gas power plants	HIFLD	Downstream	Power plants that burn gas	2075
Gas processing plants	HIFLD	Refining	Facilities designed to remove impurities and non-methane hydro-carbons from raw gas to produce pipeline-ready dry gas	619
Gas receipt delivery points	HIFLD	Downstream	Locations on gas transmission pipelines where gas is received from production systems for transmission	7,282
Intermodal pipeline freight facilities	DOT	Transportation	Transfer points between pipelines and freight	1,376
Liquified gas Import/export terminals	U.S. EIA	Transportation	Terminals used for the liquefaction of gas for transport or receipt and regasification of liquefied gas for use as gas	15
Liquefied gas storage facility	HIFLD	Storage	Above-ground storage of liquefied gas	257
Oil And gas wells	U.S. Energy information administration (EIA)	Upstream	Active oil and gas extraction wells	1198 611
Oil refineries	HIFLD	Refining	Facilities that process crude oil to generate a variety of petroleum-based products	133
Peak shaving facilities	HIFLD	Storage	Facilities that provide vaporized Liquefied Gas or Liquefied Propane Gas Air injections into gas pipelines to augment the pipeline for shortfalls in gas supplies	85
Petroleum & biomass power plants	HIFLD	Downstream	Power plants that burn a mix of petroleum and biomass	13
Petroleum & municipal solid waste power plants	HIFLD	Downstream	Power plants that burn a mix of petroleum and municipal solid waste	1
Petroleum ports	HIFLD	Transportation	Ports used to import and export petroleum products	111
Petroleum power plants	HIFLD	Downstream	Power plants that burn petroleum	993
Petroleum product terminals	U.S. EIA	Storage	Facilities primarily used for the storage, marketing, and blending of petroleum products	1390
Petroleum Pumping Stations	HIFLD	Transportation	Facilities that support the transportation of petroleum products from one location to another via a transmission pipeline.	1535
Underground gas storage facilities	DOT	Storage	Underground storage facilities for gas	396

Note : We classified power plants based on the listed fossil fuel with the highest air pollutants emissions rate per unit of fuel consumed, and whether the plant also used biomass or waste.

## 2. Methods

### 2.1. Assessment of energy infrastructure

Fossil fuel energy infrastructure data from across the contiguous United States was collected from the Energy Infrastructure Exposure Intensity and Equity Indices [EI]^3^ Database for Public Health (tables 1 and S1) [59]. The [EI]^3^ is an integrated database of spatial locations of fossil fuel energy infrastructure across all phases (i.e. downstream, midstream, and upstream) of the fossil fuel supply chain. All data were compiled from publicly available federal sources. We excluded offshore infrastructure, inactive infrastructure, and infrastructure outside of spatial bounds of census blocks in the contiguous U.S.

### 2.2. Assessment of population characteristics

We estimated populations in close proximity to energy infrastructure using a proportional population allocation (PPA) method, similar to Czolowski *et al* [58] and Michanowicz *et al* [48]. We obtained block-level population estimates and available sociodemographic measures from the 2020 Decennial Census for all states in the contiguous U.S. Subsequently, we estimated total population and their characteristics within both 800 m and 1.6 km distances of each infrastructure element. Each element of infrastructure in the [EI]3 is represented by a point; therefore, we created circular polygons for each individual element of infrastructure using radiuses of 800 m and 1.6 km Euclidian buffer distances using the reported point location of the infrastructure as the centroid. We then intersected the circular polygons with census block data and calculated representatively proportional population counts by applying the percentage of the census block within the buffer polygons to the block’s population count:

\begin{align*}&amp; {\text{Population Estimate}} \nonumber\\ &amp; \quad = \% {\text{ of the census block area with}}\;{\text{in }}800{\text{m}}\nonumber\\ &amp; \qquad \text{and }1600{\text{m buffer polygons}}\nonumber\\ &amp; \qquad *{\text{ Total population of census block}}.\end{align*}

We further estimated the population proportion using the same methods for several characteristics related to socioeconomic positioning and vulnerability: age (⩾75 years old, <5 years old), race/ethnicity (white, Black, Asian, Hispanic/Latino, American Indian or Alaskan Native [AIAN]), and urbanicity (proportion of the population living in urban areas).

### 2.3. Statistical analysis

We estimated populations residing within 800 m and 1.6 km buffers around each individual element of energy infrastructure, summarized proximate populations by the type of energy infrastructure, and compared them to the total population of the contiguous United States in 2020.

To examine disproportionate siting of fossil fuel infrastructure across non-white or Hispanic/Latino populations we conducted a log-binomial regression with a log link at the census block level. Specifically, we evaluated whether census blocks with a greater proportion of Black, Asian, Latino/Hispanic, or AIAN residents had a greater prevalence of exposure to infrastructure than majority white blocks (reference). We report the estimated prevalence ratios and 95% confidence intervals derived from heteroscedasticity-consistent robust standard errors.

For the 1.6 km buffer distance, we also quantified total populations near more than one type of infrastructure (e.g. near ⩾1 oil wells and a refinery). In addition, we compared the estimates for the population of sociodemographic groups within the buffer polygons to the total population of those sociodemographic groups. We mapped the spatial distribution of populations within 1.6 km of select emitting energy infrastructure to provide an idea of the geographic scope and calculated summary statistics for states with the highest number of residents within the 1.6 km buffer polygons.

Geospatial proximity assessment was implemented in ArcGIS Pro (version 3.2.2) [60], and statistical analysis was conducted in R version 4.5.0 [61].

## 3. Results

### 3.1. Geographic distribution of fossil fuel infrastructure

**Figure 1.**
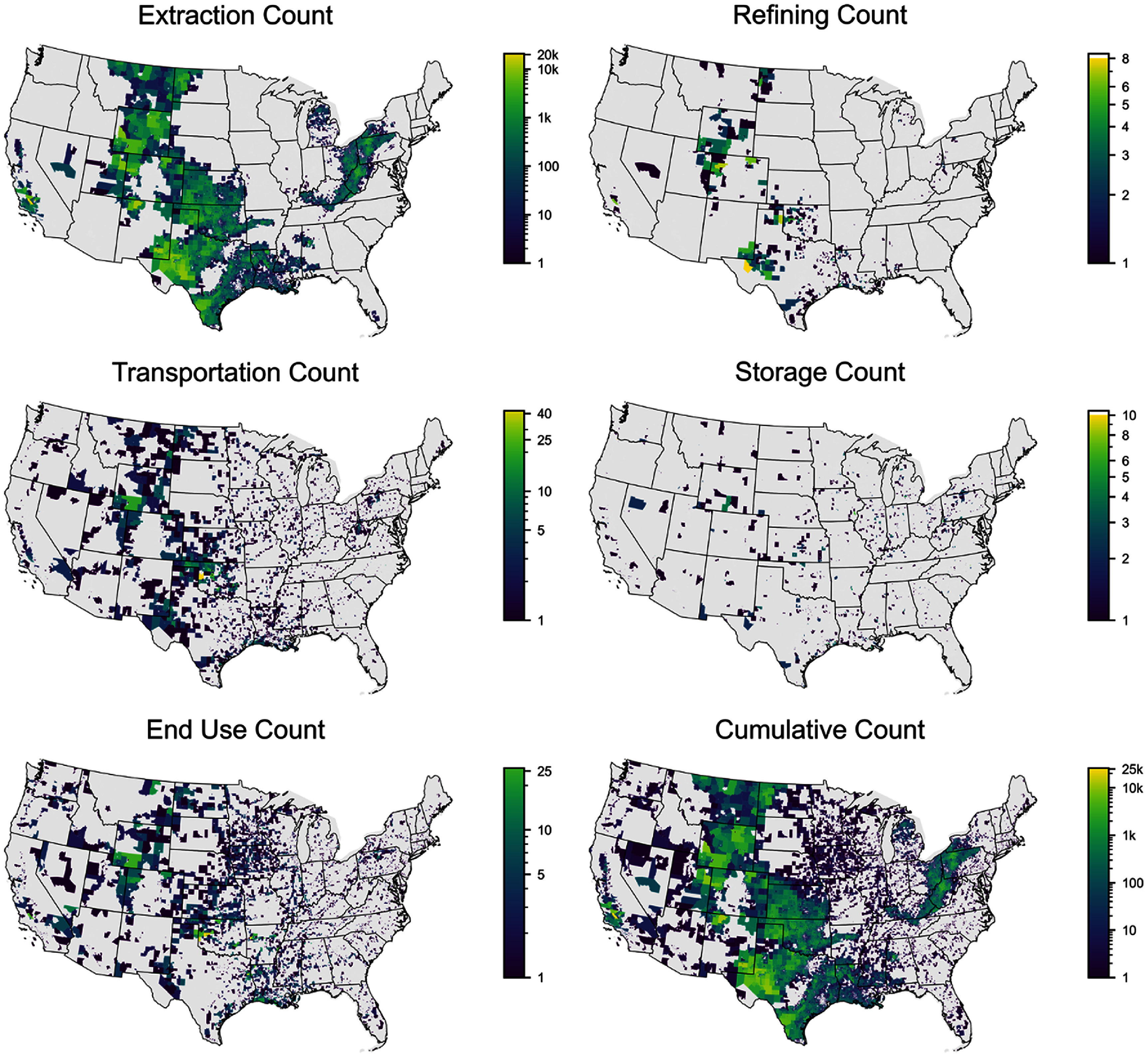
Census tract infrastructure counts by supply chain stage. Infrastructure count is symbolized on a log scale and at the census tract level for improved readability.

We observed that fossil fuel infrastructure is present in most regions of the United States, however there is substantial geographic variability in the quantity and type of infrastructure (figure 1). The Mountain West, states near the Gulf of Mexico, Appalachia, and the Great Lakes regions of the United States, and California have the most total infrastructure. Extraction and refining infrastructure is most common in in the Rocky Mountains, Gulf of Mexico, Appalachians, and California; transportation, storage, and end use infrastructure tend to be more widespread (figure 1).

### 3.2. Population near fossil fuel infrastructure

**Figure 2.**
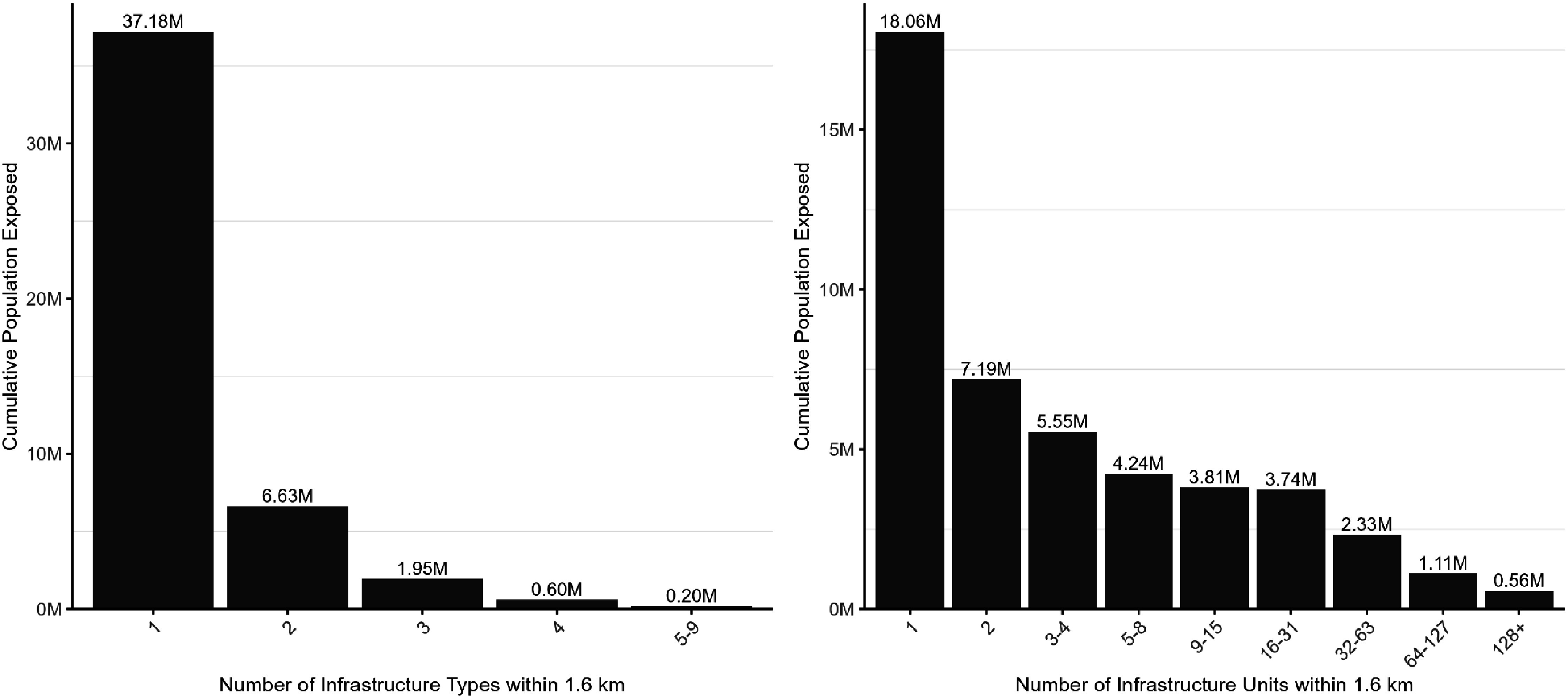
Count of population within 1.6 km of energy infrastructure by (a) number of different types of infrastructure, and (b) number of individual infrastructure elements.

We estimated that 46.6 million people reside within 1.6 km of at least one element of fossil fuel infrastructure, corresponding to about 14% of the 2020 U.S. population (figure 2, table 2). Around 18.1 million people live within 1.6 km of only one element, 12.7 million people live within 1.6 km of two to four elements, and 15.8 million people live within 1.6 km of five or more elements (figure 2). By type of infrastructure, we found 37.2 million people living within 1.6 km of one type of fossil fuel infrastructure, 6.63 million for two types, and 2.75 million people for three or more types (figure 2).

**Table 2.** Total population within 800 m and 1.6 km buffer distances from fossil fuel energy infrastructure.

	800 m			1.6 km		
Active fossil fuel energy infrastructure (*N* = 25)^a^	Population (n)^b^	Percent of contiguous US population (%)^c^	Average population by 1 element of infrastructure^d^	Population (n)^b^	Percent of contiguous US population (%)^c^	Average population by 1 element of infrastructure^d^
Oil and gas wells	10 704 429	3.3	8.9	20 266 489	6.2	17
(*N* = 1,198,611)						
Gas power plants	2199 585	0.67	1100	9341 878	2.8	4500
(*N* = 2,075)						
Petroleum power plants	2375 542	0.72	2400	7937 759	2.4	8000
(*N* = 993)						
Petroleum product terminals	1154 075	0.35	830	4989 716	1.5	3600
(*N* = 1,390)						
Gas receipt delivery points	1060 555	0.32	150	4353 678	1.3	600
(*N* = 7,282)						
Intermodal Freight Facilities—Pipelines	677 429	0.21	490	3543 972	1.1	2600
(N = 1,376)						
Petroleum pumping stations	290 858	0.088	190	1413 210	0.43	920
(*N* = 1,535)						
Liquefied gas storage facilities	165 123	0.05	640	1095 471	0.33	4300
(*N* = 257)						
Coal power plants	201 045	0.061	380	974 755	0.3	1800
(*N* = 529)						
Gas compressor stations	113 050	0.034	61	519 147	0.16	280
(*N* = 1,866)						
Peak shaving facilities	73 242	0.022	860	510 185	0.15	6000
(*N* = 85)						
Petroleum ports	62 855	0.019	570	467 748	0.14	4200
(*N* = 111)						
Oil refineries	37 983	0.012	290	319 079	0.097	2400
(*N* = 133)						
Crude Oil railroad terminals	44 617	0.014	470	250 450	0.076	2700
(*N* = 94)						
Gas processing plant	41 995	0.013	68	230 871	0.07	370
(*N* = 619)						
Underground gas storage facilities	28 404	0.0086	72	139 210	0.042	350
(*N* = 396)						
Petroleum & biomass power plants	21 314	0.0065	1600	71 683	0.022	5500
(*N* = 13)						
Surface coal mines	12 787	0.0039	38	70 537	0.021	210
(*N* = 337)						
Coal & biomass power plants	9,583	0.0029	740	36 849	0.011	2800
(*N* = 13)						
Liquefied gas import/export terminal	3,948	0.0012	260	34 027	0.01	2300
(*N* = 15)						
Gas & Biomass power plants	6,795	0.0021	340	30 611	0.0093	1500
(*N* = 20)						
Underground coal mines	6,060	0.0018	35	28 185	0.0086	160
(*N* = 174)						
Gas import/export terminals	5,156	0.0016	170	17 876	0.0054	580
(*N* = 31)						
Petroleum & municipal solid waste	722	0.00022	720	6,852	0.0021	6900
(*N* = 1)						
Gas & municipal solid waste	295	0.00009	300	4,228	0.0013	4200
(*N* = 1)						
Energy Infrastructure Supply Chain Stage^e^						
Extraction	10 716 768	3.3	8.9	20 327 652	6.2	17
(*N* = 1,199,122)						
Refining	79 951	0.024	110	547 044	0.17	730
(*N* = 752)						
Transportation	1076 114	0.33	210	5302 172	1.6	1100
(*N* = 5,028)						
Storage	1356 625	0.41	640	6156 422	1.9	2900
(*N* = 2,128)						
End Use	5701 285	1.7	520	20 931 162	6.4	1900
(*N* = 10,927)						

Cumulative	18 025 157	5.5	15	46 575 218	14	38
(*N* = 1,217,957)						

Population living within 800 m and 1.6 km buffer polygons of fossil fuel energy infrastructure.

^a^ Infrastructure compiled from the [EI]3 database. The table shows the number of each type of infrastructure (N) found within the contiguous US.

^b^ Population of census blocks from the 2020 Decennial Census intersecting with 800 m and 1.6 km radius buffer polygons around energy infrastructure.

^c^ Percent of the contiguous United States population in 2020 (*n* = 329,260,619).

^d^ Rounded to two significant figures.

^e^ Fossil fuel energy infrastructures are grouped into categories dependent on their place along the energy supply chain stage. End use infrastructure includes gas receipt facilities, petroleum power plants, gas power plants, coal power plants, coal/biomass power plants, gas/biomass power plants, petroleum/biomass power plants, petroleum/municipal solid waste power plants, and gas/municipal solid waste power plants. Extraction infrastructure includes underground coal mines, surface coal mines, and oil and gas wells. Storage infrastructure includes gas storage facilities, peak shaving facilities, petroleum product terminals, and liquefied gas storage facilities. Transportation infrastructure includes gas import/export facilities, gas compressor stations, petroleum ports, petroleum pumps, crude oil rail terminals, and liquefied gas import/export facilities. Refining infrastructure includes gas processing facilities and oil refineries.

Eight types of fossil fuel energy infrastructure have more than a million people residing within 1.6 km (table 2), with oil and gas wells showing the most people who reside within 1.6 km (table 2). Five types of fossil fuel energy infrastructure have over a million people living within 800 m—again, oil and gas wells have the most (table 2). There are 16 types of infrastructure with an average of more than 1,000 people living within 1.6 km of each element of infrastructure. Petroleum power plants are the highest, with an average of ∼8,000 people within 1.6 km of each power plant (table 2). Oil and gas wells have the fewest, with an average of 17 people living within 1.6 km of each individual well (table 2).

**Figure 3.**
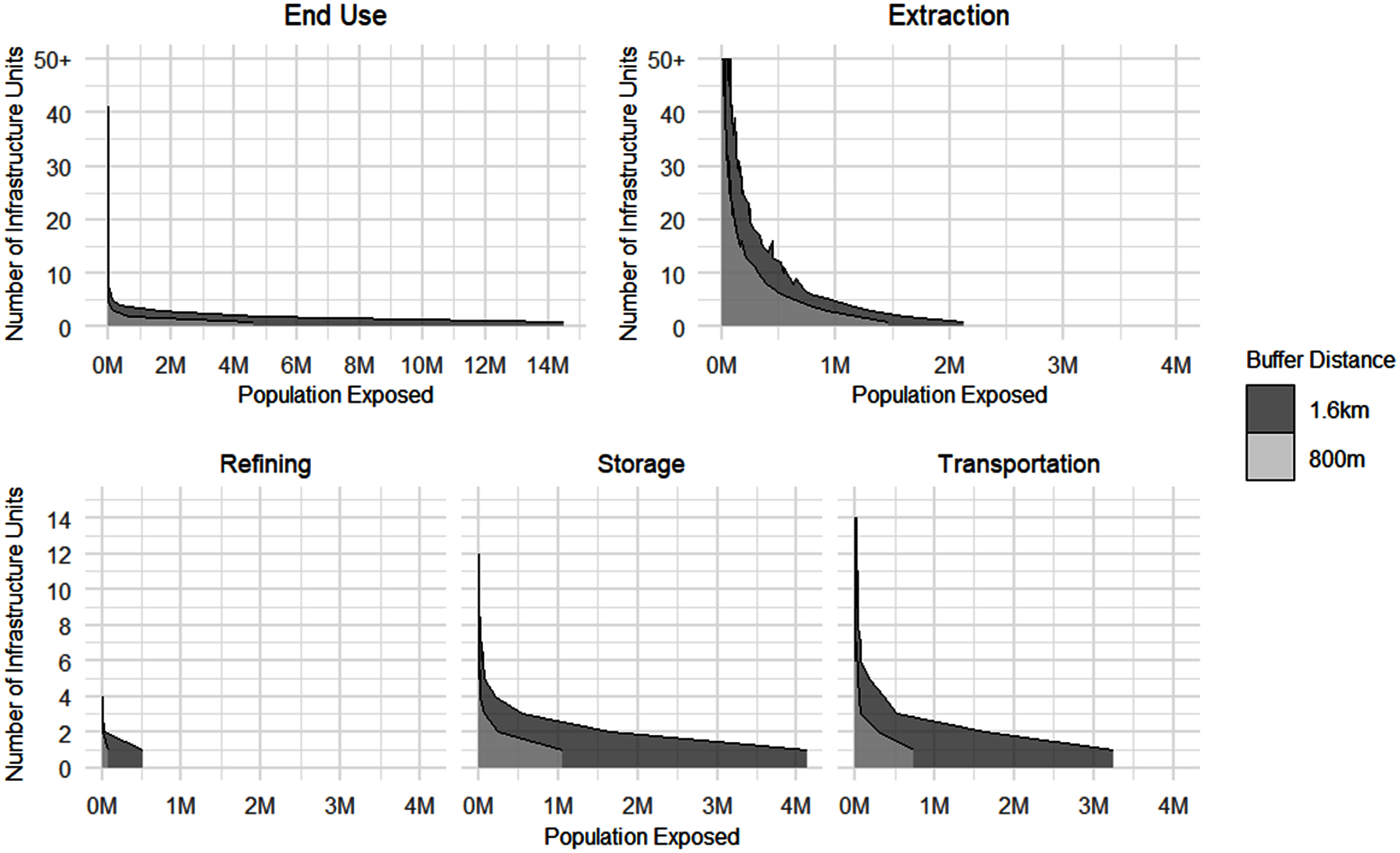
Density chart of number of infrastructure units by count of population (in millions) within 1.6 km and 800 m, by stage of supply chain.

Across supply chain stages, end use infrastructure has the most people residing within 1.6 km, followed by extraction (table 2). Similarly, extraction has the largest population within 800 m, followed by end use. When normalized per element of infrastructure, storage has the most people within both 1.6 km and 800 m distances, followed by end use. Extraction has the least, with an average of 17 people within 1.6 km of each element of infrastructure and 8.9 people within 800 m. Distributions of populations near each individual unit of infrastructure vary quite substantially, and are very heavily right-skewed—many individual infrastructure units have zero or a few people nearby, especially extraction, and a few infrastructure units having half a million or more people living nearby (figure 3).

### 3.3. Disparities between racial and ethnic groups near fossil fuel infrastructure

Across all fossil fuel energy infrastructure, 16.4% of the 2020 U.S. population who identified as Hispanic and/or Latino are within 1.6 km of at least one element of fossil fuel infrastructure in the U.S., followed by American Indian/Alaska Native at 16.0%, Asian at 15.4%, and 14.2% of populations identified as Black (table 3). However, this pattern varies across the supply chain. Populations living within 1.6 km of extraction infrastructure are disproportionately American Indian/Alaska Native; populations identifying as Hispanic and/or Latino are most disproportionately those within 1.6 km of storage, transportation, and refining; populations identifying as Asian are most disproportionately within 1.6 km of end use (table 3). Similar patterns within groups exist at 800 m (table S3).

**Table 3.** Total population within a 1.6 km buffer distance from fossil fuel infrastructure by race/ethnicity group.

	Total population	White	Black	Asian	Hispanic/Latino	American Indian/Alaskan native
Active fossil fuel energy infrastructure (*N* = 25) ^a^	N	n (% of Population Group) ^b^				
Oil and gas wells	20 266 489	13 727 691 (6.7)	1783 597 (4.3)	733 252 (3.8)	3839 813 (6.2)	310 615 (8.6)
Gas power plants	9341 878	4702 868 (2.3)	1224 765 (3.0)	1060 124 (5.5)	2370 227 (3.8)	93 728 (2.6)
Petroleum power plants	7937 759	3787 622 (1.9)	1440 347 (3.5)	806 190 (4.2)	1879 556 (3.0)	72 272 (2.0)
Petroleum product terminals	4989 716	2394 384 (1.2)	881 106 (2.1)	305 134 (1.6)	1451 937 (2.3)	61 704 (1.7)
Gas receipt delivery points	4353 678	2938 501 (1.4)	548 958 (1.3)	174 293 (0.903)	604 572 (0.98)	43 823 (1.2)
Intermodal freight facilities—pipelines	3543 972	1666 508 (0.82)	600 318 (1.5)	237 675 (1.2)	1065 061 (1.7)	43 292 (1.2)
Petroleum pumping stations	1413 210	743 108 (0.37)	188 903 (0.46)	70 095 (0.36)	433 179 (0.70)	20 577 (0.57)
Liquefied gas storage facilities	1095 471	467 130 (0.23)	169 664 (0.41)	87 465 (0.45)	384 736 (0.62)	13 938 (0.39)
Coal power plants	974 755	658 295 (0.32)	108 589 (0.26)	46 872 (0.24)	139 903 (0.23)	10 157 (0.28)
Gas compressor stations	519 147	348 485 (0.17)	58 519 (0.14)	25 769 (0.13)	76 635 (0.12)	6985 (0.19)
Peak shaving facilities	510 185	260 364 (0.13)	74 988 (0.18)	33 426 (0.17)	141 580 (0.23)	3815 (0.11)
Petroleum ports	467 748	248 705 (0.12)	75 169 (0.18)	15 947 (0.083)	123 582 (0.20)	5949 (0.16)
Oil refineries	319 079	151 758 (0.075)	40 243 (0.098)	11 885 (0.062)	122 666 (0.20)	6387 (0.18)
Crude oil railroad terminals	250 450	121 334 (0.060)	35 892 (0.087)	6619 (0.034)	91 011 (0.15)	6683 (0.19)
Gas processing facilities	230 871	93 225 (0.046)	28 455 (0.069)	26 973 (0.14)	82 227 (0.13)	4711 (0.13)
Underground gas storage facilities	139 210	100 152 (0.049)	11 581 (0.028)	8184 (0.042)	15 891 (0.026)	775 (0.021)
Petroleum & biomass power plants	71 683	50 283 (0.025)	6618 (0.016)	7845 (0.041)	5097 (0.0082)	426 (0.012)
Surface coal mines	70 537	63 504 (0.031)	2096 (0.0051)	342 (0.0018)	2904 (0.0047)	491 (0.014)
Coal & biomass plants	36 849	28 612 (0.014)	3,734 (0.0091)	891 (0.0046)	2018 (0.0033)	437 (0.012)
Liquefied gas Import/export terminal	34 027	13 697 (0.0067)	2203 (0.0054)	1482 (0.0077)	19 225 (0.031)	130 (0.0036)
Gas & biomass power plants	30 611	21 050 (0.0100)	3412 (0.0083)	1324 (0.0069)	3340 (0.0054)	525 (0.015)
Underground coal mines	28 185	26 399 (0.013)	359 (0.000 87)	108 (0.000 56)	451 (0.000 73)	40 (0.0011)
Gas import/export terminals	17 876	10 086 (0.0050)	1,945 (0.0047)	272 (0.0014)	6,101 (0.0099)	457 (0.013)
Petroleum & municipal solid waste power plants	6852	5,039 (0.0025)	614 (0.0015)	402 (0.0021)	572 (0.00092)	76 (0.0021)
Gas & municipal solid Waste power plants	4228	3,697 (0.0018)	139 (0.00034)	53 (0.00028)	146 (0.00024)	21 (0.00059)

Energy Infrastructure Supply Chain Stage ^c^						
Extraction	20 327 652	13 782 318 (6.8)	1785 454 (4.3)	733 535 (3.8)	3842 715 (6.2)	311 073 (8.6)
Refining	547 044	242 556 (0.12)	68 584 (0.17)	38 840 (0.20)	204 625 (0.33)	11 077 (0.31)
Transportation	5302 172	2670 954 (1.3)	805 508 (2.0)	315 348 (1.6)	1540 630 (2.5)	70 346 (1.9)
Storage	6156 422	2927 921 (1.4)	1052 087 (2.6)	395 328 (2.0)	1835 003 (3.0)	75 958 (2.1)
End Use	20 931 162	11 138 740 (5.5)	3104 114 (7.6)	1925 047 (10.0)	4657 153 (7.5)	205 299 (5.7)

Cumulative	46 575 218	27 412 890 (13.5)	5822 908 (14.2)	2977 612 (15.4)	10 126 951 (16.4)	579 395 (16.0)

Population by race/ethnicity group population within 1.6 km buffer polygons of fossil fuel energy infrastructure. U.S. residents self-identify themselves as part of race/ethnicity groups in the Census.

^a^ Infrastructure compiled from the [EI]3 database.

^b^ Population of race/ethnicity groups in census blocks from the 2020 Decennial Census intersecting with 1.6 km radius buffer polygons around fossil fuel energy infrastructure. The percentage is out of the total population of the race/ethnicity group in the 2020 Decennial Census.

^c^ Fossil fuel energy infrastructures are grouped into categories dependent on their place along the energy supply chain stage. End use infrastructure includes gas receipt facilities, petroleum power plants, gas power plants, coal power plants, coal/biomass power plants, gas/biomass power plants, petroleum/biomass power plants, petroleum/municipal solid waste power plants, and gas/municipal solid waste power plants. Extraction infrastructure includes underground coal mines, surface coal mines, and oil and gas wells. Storage infrastructure includes gas storage facilities, peak shaving facilities, petroleum product terminals, and liquefied gas storage facilities. Transportation infrastructure includes gas import/export facilities, gas compressor stations, petroleum ports, petroleum pumps, crude oil rail terminals, and liquefied gas import/export facilities. Refining infrastructure includes gas processing facilities and oil refineries.

Populations in predominately non-white or Hispanic/Latino census blocks are disproportionately within 1.6 km of refining, transportation, storage, and end use infrastructure as compared to predominately white blocks (tables 3 and S2). The highest disparities include: a 3.05 (95% CI: 2.64, 3.51) times higher proportion in predominantly Black census blocks near gas processing facilities, a 4.15 (95% CI: 3.52, 4.89) times higher proportion in predominantly Asian census blocks near gas processing facilities, a 3.42 (95% CI: 3.23, 3.63) times higher proportion in predominantly Hispanic/Latino census blocks near liquefied gas import/export terminals, and a 2.49 (95% CI: 2.06, 3.00) times higher proportion in predominantly American Indian/Alaska Native census blocks near oil refineries (tables 3 and S2). For all infrastructure, compared to predominantly white census blocks, there is a 1.19 (95% CI: 1.17, 1.20) times higher exposed population in blocks that are predominantly Black, a 1.18 (95% CI: 1.15, 1.20) times higher proportion in blocks that are predominantly Asian, and a 1.33 (95% CI: 1.32, 1.35) times higher proportion in blocks that are predominantly Hispanic/Latino (table S2). Broadly, we find similar trends at the 800 m distance (tables S3 and S4).

### 3.4. Disparities in fossil fuel exposures by age and urbanicity

We estimate that 2.6 million children under the age of 5 and 2.9 million adults over the age of 75 were within 1.6 km of fossil fuel energy infrastructure. Across all supply chain stages, a slightly higher proportion of children under the age of 5 are within 1.6 km of fossil fuel infrastructure than adults over the age of 75 (table 4). This was also true at 800 m, except for refining (table S4). More than 90% of the populations within 1.6 km of end use, refining, transportation, and storage infrastructure are urban. Approximately two thirds of the population within 1.6 km of extraction infrastructure is urban, almost entirely driven by oil and gas wells.

**Table 4.** Total population within a 1.6 km buffer distance from fossil fuel infrastructure by age group and urbanicity.

	Total population within 1.6 km	Under age 5	75 Years of age and older	Urban population
Active fossil fuel energy infrastructure (*N* = 25) ^a^	N	n (% of Population Group) ^b^		
Oil and gas wells	20 266 489	1158 540 (6.3)	1383 311 (6.1)	13 523 003 (5.1)
Gas power plants	9341 878	470 032 (2.6)	504 854 (2.2)	9104 188 (3.5)
Petroleum power plants	7937 759	427 869 (2.3)	498 487 (2.2)	7504 010 (2.8)
Petroleum product terminals	4989 716	292 414 (1.6)	272 627 (1.2)	4869 953 (1.8)
Gas receipt delivery points	4353 678	254 280 (1.4)	298 884 (1.3)	3271 095 (1.2)
Intermodal freight facilities—pipelines	3543 972	209 966 (1.1)	193 799 (0.86)	3453 127 (1.3)
Petroleum pumping stations	1413 210	85 205 (0.47)	79 801 (0.35)	1267 284 (0.48)
Liquefied gas storage facilities	1095 471	66 316 (0.36)	55 691 (0.25)	1059 619 (0.40)
Coal power plants	974 755	46 964 (0.26)	52 166 (0.23)	918 705 (0.35)
Gas compressor stations	519 147	28 024 (0.15)	34 524 (0.15)	330 264 (0.13)
Peak shaving facilities	510 185	30 156 (0.17)	27 040 (0.12)	497 826 (0.19)
Petroleum ports	467 748	24 186 (0.13)	25 031 (0.11)	462 863 (0.18)
Oil refineries	319 079	19 935 (0.11)	17 157 (0.076)	308 828 (0.12)
Crude oil railroad terminals	250 450	16 054 (0.088)	13 301 (0.059)	231 439 (0.088)
Gas processing facilities	230 871	12 044 (0.066)	15 156 (0.067)	189 205 (0.072)
Underground gas storage facilities	139 210	7507 (0.041)	9171 (0.041)	95 139 (0.036)
Petroleum & biomass power plants	71 683	2442 (0.013)	2171 (0.0096)	67 492 (0.026)
Coal surface mines	70 537	3404 (0.019)	6200 (0.028)	25 471 (0.0097)
Coal & biomass power plants	36 849	1979 (0.011)	3076 (0.014)	35 868 (0.014)
Liquefied gas import/export terminal	34 027	1748 (0.0096)	2676 (0.012)	33 860 (0.013)
Gas & biomass power plants	30 611	1722 (0.0094)	2314 (0.0100)	28 103 (0.011)
Coal underground mines	28 185	1423 (0.0078)	2426 (0.011)	8,869 (0.0034)
Gas import/export terminals	17 876	973 (0.0053)	1573 (0.0070)	15 364 (0.0058)
Petroleum & municipal solid waste power plants	6852	424 (0.0023)	357 (0.0016)	6816 (0.0026)
Gas & municipal solid waste power plants	4228	235 (0.0013)	413 (0.0018)	4218 (0.0016)

Energy Infrastructure Supply Chain Stage ^C^				
Extraction	20 327 652	1161 476 (6.4)	1388 969 (6.2)	13 548 325 (5.1)
Refining	547 044	31 804 (0.17)	32 151 (0.14)	495 351 (0.19)
Transportation	5302 172	309 631 (1.7)	301 560 (1.3)	4886 357 (1.9)
Storage	6156 422	363 742 (2.0)	333 875 (1.5)	5957 190 (2.3)
End Use	20 931 162	1114 363 (6.1)	1256 293 (5.6)	19 238 539 (7.3)

Cumulative	46 575 218	2596 725 (14.2)	2940 371 (13.0)	37 941 129 (14.4)

Age-group and urban populations within 1.6 km radius buffer polygons of fossil fuel energy infrastructure. Populations in bold are higher than the nationwide proportion in that age group or urbanicity group. According to the 2020 US Decennial Census, in the contiguous US there were 18 274 779 children under 5 years of age (5.6% of total U.S. population), 22 534 060 adults 75 years of age and over (6.8%), and 263 420 610 living in urban areas (80%).

^a^ Infrastructure compiled from the [EI]^3^ database.

^b^ Population of race/ethnicity groups in census blocks from the 2020 Decennial Census within 1.6 km radius buffer polygons around fossil fuel energy infrastructure. The percentage is out of the total population of the age group/urban population in the 2020 Decennial Census.

^c^ Fossil fuel energy infrastructures are grouped into categories dependent on their place along the energy supply chain stage. End use infrastructure includes gas receipt facilities, petroleum power plants, gas power plants, coal power plants, coal/biomass power plants, gas/biomass power plants, petroleum/biomass power plants, petroleum/municipal solid waste power plants, and gas/municipal solid waste power plants. Extraction infrastructure includes underground coal mines, surface coal mines, and oil and gas wells. Storage infrastructure includes gas storage facilities, peak shaving facilities, petroleum product terminals, and liquefied gas storage facilities. Transportation infrastructure includes gas import/export facilities, gas compressor stations, petroleum ports, petroleum pumps, crude oil rail terminals, and liquefied gas import/export facilities. Refining infrastructure includes gas processing facilities and oil refineries.

### 3.5. Regional variation in fossil fuel infrastructure near populations

**Figure 4.**
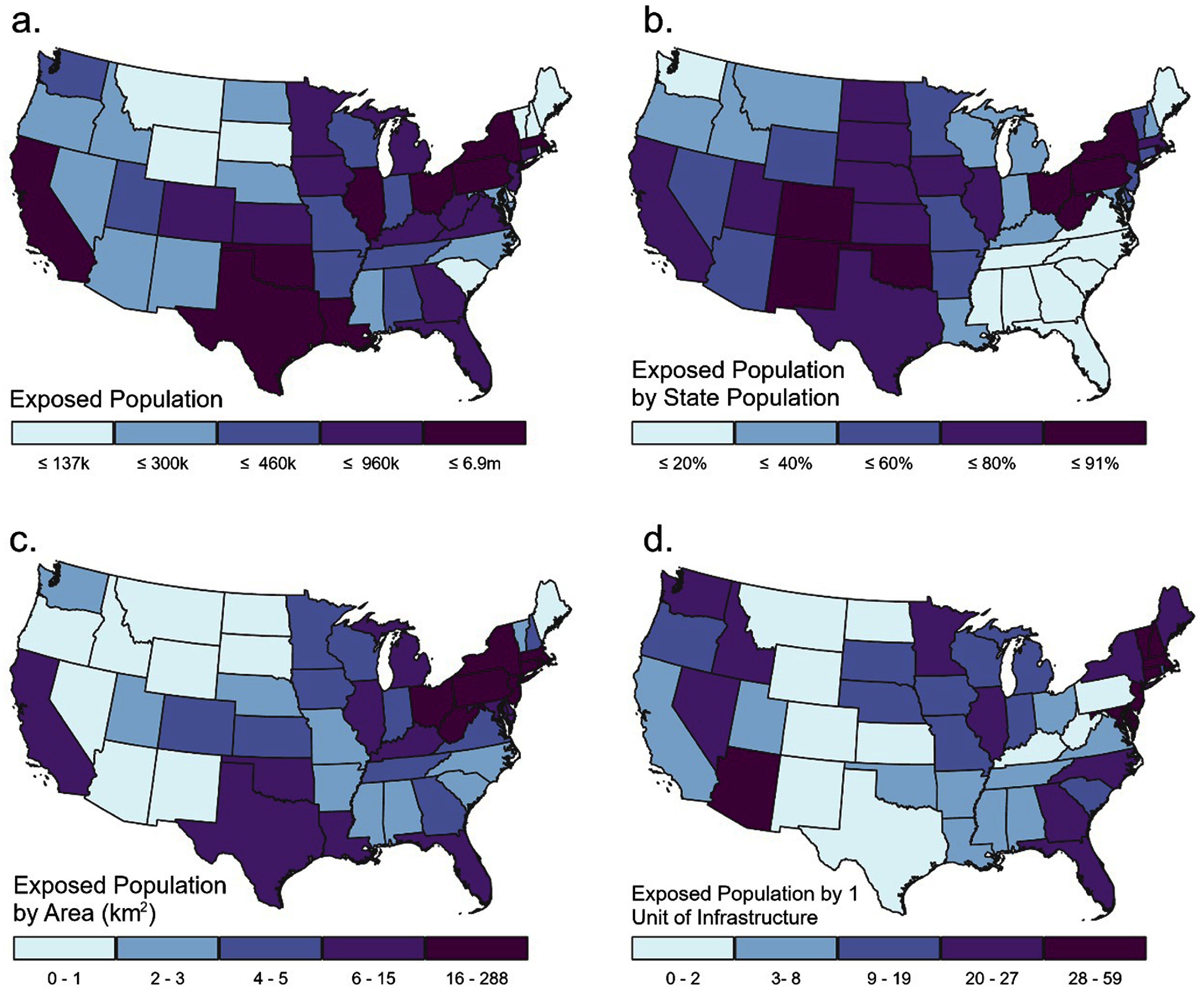
State population within 1.6 km of energy infrastructure (a) by absolute count, (b) by percentage of state population, (c) per state area, and (d) per unit of energy infrastructure in that state. All four map are symbolized using quartiles.

New York, Massachusetts, Pennsylvania, Ohio, Illinois, Oklahoma, Louisiana, Texas, and California had the highest populations within 1.6 km of fossil fuel infrastructure (figure 4(a)). Many states in the Midwest and Mountain West have over 60% of their population within 1.6 km of fossil fuel infrastructure (figure 4(b)). When normalized by state area, states in the Mid-Atlantic and southern New England have the highest populations within 1.6 km of fossil fuel infrastructure (>16 people per km^2^), while states in the Mountain West are lowest (>1 person per km^2^) (figure 4(c)). When normalized per unit of infrastructure, Arizona and some states in the Northeast have highest populations within 1.6 km of fossil fuel infrastructure (>20 people on average) (figure 4(d)).

**Figure 5.**
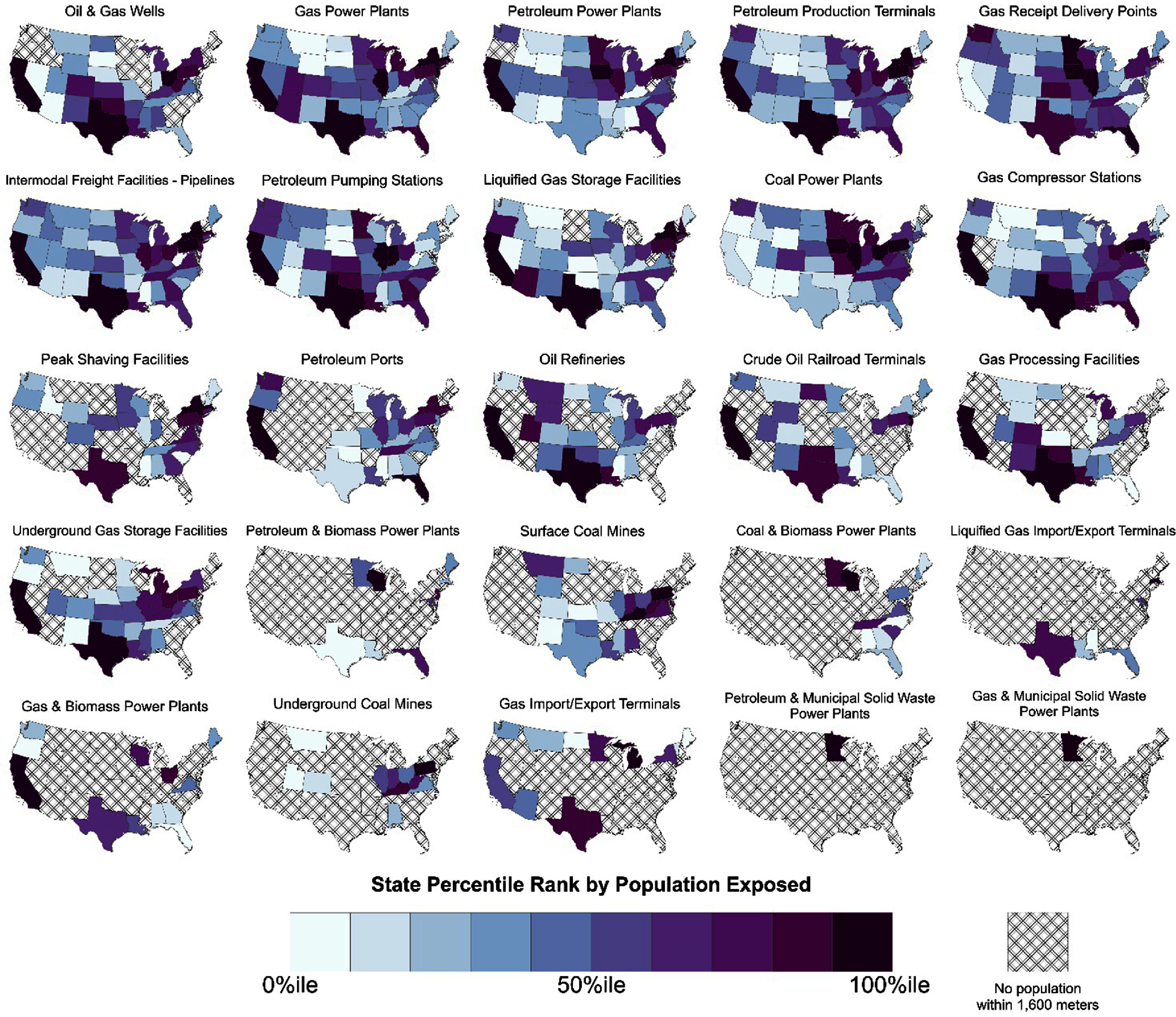
State populations within 1.6 km of fossil fuel energy infrastructure, ranked by decile. Infrastructure types are ordered according to the total population within 1.6 km of that type of infrastructure.

The infrastructure driving these trends varies by state. End use infrastructure is more geographically dispersed (figure 5). The geographic patterns of midstream infrastructure and nearby populations were less clear. States with the most people near oil refining and gas processing also had the most people near extraction (e.g. Texas, California). Most types of storage infrastructure were present in the majority of states and had people residing within 1.6 km, except underground gas storage facilities, which followed spatial patterns more similar to oil and gas wells (figure 5).

## 4. Discussion

We find that approximately 46.6 million people, or 14.1% of the contiguous U.S. population, live within 1.6 km (∼1 mile) of at least one element of fossil fuel infrastructure. We estimated that 28.5 million people live near more than one element of fossil fuel infrastructure, potentially experiencing higher exposures. Moreover, an estimated 9.38 million people live near more than one type of fossil fuel infrastructure, potentially exposed to a greater mixture of hazards. Persistently marginalized racial and ethnic groups are disproportionately residing within close proximity to many types of fossil fuel infrastructure across all stages of the energy supply chain.

Populations living near each type of infrastructure varied by more than four orders of magnitude, and average population living near each element of infrastructure varied by more than three orders of magnitude. Eight types of infrastructure had over a million people living within 1.6 km, and 16 types of infrastructure had more than 1,000 people living within 1.6 km of each individual element of infrastructure. For some infrastructure types and some states, the number of people exposed was driven by the ubiquitousness of a type of infrastructure (e.g. oil and gas wells—20.3 million people within 1.6 km but an average of 17 people per well); for others, it was driven by a few elements of infrastructure sited where a high number of people live near each element of infrastructure (e.g. petroleum power plants with 8,000 people living within 1.6 km of the average power plant).

There were very different exposure patterns across the different supply chain stages. Extraction infrastructure had the second most people within 1.6 km (∼20.3 million) and the most people within 800 m (∼10.7 million), but the lowest number of people residing near each individual infrastructure element. This indicates that populations living near extraction infrastructure is driven by its pervasiveness. In contrast, end use was the supply chain stage with the most people—around 20.9 million—living within 1.6 km of at least one element of infrastructure, largely driven by a small number of individual elements of infrastructure with high populations nearby. Storage infrastructure had the highest average population living nearby per element of infrastructure but relatively few people living nearby in aggregate, since storage was the second least common type of infrastructure.

Nuances of relationships between populations and fossil fuel energy infrastructure varies by state. Texas, Pennsylvania, Ohio, and California have very high total populations residing near infrastructure. However, Texas and California have lower populations per total area and lower percentages of total population near fossil fuel energy infrastructure than Ohio and Pennsylvania. This reflects Ohio and Pennsylvania having higher proportions of their populations within 1.6 km of an element of infrastructure than California and Texas. Vermont, New Hampshire, Massachusetts, and Maryland have high populations near each element of infrastructure, despite lower total populations near infrastructure. This result indicates that those states have energy infrastructure sited in populated areas, and little to no extraction infrastructure.

Our findings of racial/ethnic disparities are consistent with previous literature, revealing similar trends for most types of energy infrastructure across all supply chain stages [11, 18, 33, 42, 62]. Populations identifying as Hispanic/Latino had the highest disparities in exposure, and were the most disproportionately exposed group for refining, transportation, and storage. The populations most burdened with end-use infrastructure were those identifying as Asian. For extraction, the group with the highest proportion living nearby was American Indian/Alaska Native. We did not find any substantial disparities in terms of ages of populations, but do find that fossil fuel infrastructure is near both rural and urban populations, with little variation by region.

### 4.1. Limitations

This analysis likely underestimates population counts since it measures population from the infrastructure centroid. Many elements of fossil fuel energy infrastructure are full facilities, potentially with multiple buildings and a ‘fence line’ boundary and are likely better represented as polygons encompassing the full facility footprint. Some fossil fuel energy infrastructure exists below the land surface (e.g., underground gas storage facilities, coal mines), so the most relevant point of exposure may be single or multiple elements of infrastructure on the surface (e.g. well heads, mine entrances) [47]. This may particularly influence our counts of populations near in our end use, storage, and refining infrastructure, since these infrastructure types can have large footprints above ground or underground. Due to data availability, our underlying data set—the [EI]3—does not include some relevant elements of fossil fuel energy infrastructure, such as pipelines (e.g. gas utility distribution, gathering pipelines), ancillary equipment (e.g. water separators, holding tanks, gathering compressors), industrial users, and end use components in residential and commercial buildings (e.g. stoves, furnaces, and other fossil fuel-consuming appliances). Since federal infrastructure data quality varies, the [EI]3 dataset may substantially undercount some elements of energy infrastructure (e.g. compressor stations [6]). Our research does not represent the entirety of fossil fuel supply chains since the underlying dataset is known to be missing these relevant pieces of infrastructure. Despite this, it is an advancement from previous work, expanding the scope of infrastructure types being assessed.

Our study used U.S. Census data at the highest resolution available—census block—with a PPA method, which may misallocate populations near each piece of infrastructure [48]. However, since our buffer distances are relatively large (i.e. 800 m and 1.6 km), we expect the degree of underestimation introduced to be relatively small and nondifferential [48]. Similarly, while there is uncertainty associated with the precision of infrastructure location, manual assessment of a subset of each infrastructure dataset revealed infrequent spatial errors of substantial magnitude, so we expect this to have negligible impact on our analysis. We were unable to estimate disproportionate exposure related to other components of socioeconomic status, such as income or educational attainment, since that data is unavailable at the census block level.

Although 800 m and 1.6 km buffers are consistent with some previous literature [58, 63], this distance is not necessarily health-relevant in all contexts. Larger distances than the ones we used here would result in larger populations being categorized as near this infrastructure, which may better reflect the true reach of the associated emissions. A wide range of distances have been proposed or used in a variety of contexts—for instance, a safety-oriented perspective may suggest a 200 m radius for oil and gas wells based on heat flux during a well explosion [64] while an air pollution focus would argue that oil and gas production has continental reach [6, 65]. Therefore, our results do not represent the full scope of physical and chemical processes leading to emission of a pollutant or creation of a potential hazard, and population exposure to that hazard.

### 4.2. Policy implications and future work

This study shows that the sociodemographic disparities are persistent across the entire fossil fuel supply chain, and that many people reside in close proximity to fossil fuel infrastructure. The differences in exposure distribution across supply chain stages (i.e. ubiquitousness of infrastructure vs. siting in urban areas) may indicate that strategies that effectively control exposure to hazards may differ across supply chain phases. These findings can be used to refine existing state policies, for example those requiring that new oil and gas wells must be a certain distance from certain types of buildings—a family of policies commonly known as ‘setbacks’ [2, 66]. Setback regulations can be placed on more types of fossil fuel infrastructure across the supply chain to prevent or reduce community exposures, especially any additional burdens on persistently marginalized populations [2, 66]. Findings could be incorporated into ‘reverse setbacks’ regulations which restrict construction of new buildings near fossil fuel infrastructure to limit population encroachment [48, 66–68]. To further reduce the burden on local communities, nearby populations and the possibility of exacerbating disparities could be included in the decision-making process around new construction of fossil fuel energy infrastructure, including decisions around siting and granting permits.

Our results lay the groundwork for future research in a variety of domains. A growing body of epidemiologic research has examined the population health implications of residing near oil and gas extraction, power plants, and compressor stations [20–24, 26–28, 45]; this work could be expanded to all fossil fuel energy infrastructure and all relevant health outcomes, and could expand to include communities exposed to mixtures of different types of infrastructure. Building upon our proximity modeling, future exposure assessment work could integrate environmental modeling to better estimate populations exposures to air and water pollution from these facilities, along with the potential noise, light, and induced traffic hazards, to better differentiate which populations are exposed to which hazards [6, 12–14, 51, 57, 69, 70]. To fully characterize the health impacts in exposed communities, especially those near multiple types of energy infrastructure, a framework such as Cumulative Impact Assessment [19, 71], could be implemented to better understand and quantify the multiple hazards and broader impacts that energy infrastructure poses on nearby communities.

The information and methods here could be useful for ensuring that the energy transition is implemented in an equitable manner. Populations near both legacy and new infrastructure could be an important decision metric for planning, siting, and design of energy transition projects, alongside other metrics, such as cost and carbon emissions avoided. Even if the economy successfully transitions to renewable energy, and emissions of greenhouse gases and air pollutants are largely mitigated, there may still be substantial populations exposed to hazards related to legacy infrastructure. For instance, abandoned oil wells, gas wells, coal mines still emit air pollution at levels that may harm human health [72–74]; similar exposures may be present around other abandoned infrastructure. Novel energy and climate infrastructure, such as carbon capture and sequestration, could potentially expose populations to associated health and safety hazards [75, 76]. Some types of renewable energy, such as use of hydrogen as a combustion fuel, may expose local communities to air pollutants, especially NO*_x_*, and potentially pose other hazards [77–79]. Many of these hazards are attenuated by distance [80], so prioritizing decommissioning fossil fuel energy infrastructure with large populations nearby—especially vulnerable populations—may be an important strategy for equitable implementation of the energy transition. Similarly, siting new energy or carbon capture and sequestration infrastructure away from vulnerable populations, especially if it poses hazards, may also be an important strategy. Including populations nearby along with other decision-relevant metrics, such as cost, capacity, and greenhouse gas emissions avoided or carbon sequestered, could be a useful way to incorporate equity of siting and health of local communities in decision-making around the energy transition [81]. Considering local communities hosting this infrastructure in such as way may help to ensure that the energy transition occurs in a way that is both healthy and just [82–86].

## Data Availability

The data that support the findings of this study are openly available at the following URL/DOI: https://doi.org/10.7910/DVN/KLUSQB [59].
